# Scaffolded and annotated nuclear and organelle genomes of the North American brown alga *Saccharina latissima*

**DOI:** 10.3389/fgene.2025.1494480

**Published:** 2025-05-14

**Authors:** Kelly DeWeese, Gary Molano, Sara Calhoun, Anna Lipzen, Jerry Jenkins, Melissa Williams, Christopher Plott, Jayson Talag, Jane Grimwood, Jean-Luc Jannink, Igor V. Grigoriev, Jeremy Schmutz, Charles Yarish, Sergey Nuzhdin, Scott Lindell

**Affiliations:** ^1^ Department of Molecular and Computational Biology, University of Southern California, Los Angeles, CA, United States; ^2^ US Department of Energy Joint Genome Institute, Lawrence Berkeley National Laboratory, Berkeley, CA, United States; ^3^ Genome Sequencing Center, HudsonAlpha Institute for Biotechnology, Huntsville, AL, United States; ^4^ Arizona Genomics Institute, School of Plant Sciences, University of Arizona, Tucson, AZ, United States; ^5^ US Department of Agriculture, Agricultural Research Service (USDA-ARS), Ithaca, NY, United States; ^6^ Section On Plant Breeding and Genetics, School of Integrative Plant Sciences, Cornell University, Ithaca, NY, United States; ^7^ Department of Plant and Microbial Biology, University of California Berkeley, Berkeley, CA, United States; ^8^ Department of Ecology and Evolutionary Biology, University of Connecticut, Stamford, CT, United States; ^9^ Applied Ocean Physics and Engineering Department, Woods Hole Oceanographic Institution, Woods Hole, MA, United States

**Keywords:** Saccharina latissima, sugar kelp, genome, comparative genomics, genomic breeding, brown macroalga, kelp aquaculture

## Abstract

Increasing the genomic resources of emerging aquaculture crop targets can expedite breeding processes as seen in molecular breeding advances in agriculture. High quality annotated reference genomes are essential to implement this relatively new molecular breeding scheme and benefit research areas such as population genetics, gene discovery, and gene mechanics by providing a tool for standard comparison. The brown macroalga *Saccharina latissima* (sugar kelp) is an ecologically and economically important kelp that is found in both the northern Pacific and Atlantic Oceans. Cultivation of *Saccharina latissima* for human consumption has increased significantly this century in both North America and Europe, and its single blade morphology allows for dense seeding practices used in the cultivation of its Asian sister species, *Saccharina japonica*. While *Saccharina latissima* has potential as a human food crop, insufficient information from genetic resources has limited molecular breeding in sugar kelp aquaculture. We present scaffolded and annotated *Saccharina latissima* nuclear and organelle genomes from a female gametophyte collected from Black Ledge, Groton, Connecticut. This *Saccharina latissima* genome compares well with other published kelp genomes and contains 218 scaffolds with a scaffold N50 of 1.35 Mb, a GC content of 49.84%, and 25,012 predicted genes. We also validated this genome by comparing the synteny and completeness of this *Saccharina latissima* genome to other kelp genomes. Our team has successfully performed initial genomic selection trials with sugar kelp using a draft version of this genome. This *Saccharina latissima* genome expands the genetic toolkit for the economically and ecologically important sugar kelp and will be a fundamental resource for future foundational science, breeding, and conservation efforts.

## 1 Introduction

The demand for sustainable farming resources is increasing due to the combination of rising global temperatures (Abbass et al., 2022), increasing population levels (Pimentel, 1991), and decreasing amounts of arable land (Thaler et al., 2021). Increasing global food production can help raise the carrying capacity of Earth despite these environmental challenges escalating in the 21st century (Hopfenberg, 2003). One potential sustainable resource solution is to increase the amount of biomass produced in the ocean by deploying open ocean kelp farms, as kelp grows quickly and requires no arable land, freshwater, herbicides, or fertilizers (Kim et al., 2019a). Kelp are haplodiplontic brown macroalgae (North, 1987; Diehl et al., 2024) that provide vital ecosystem services, including habitat creation and primary production (Eger et al., 2023), that sustain some of the ocean’s most diverse communities (Dayton, 1985; Steneck et al., 2002; Wernberg et al., 2018). The emergence of the kelp lineage (Laminariales) is estimated to have occurred over 80 million years ago (Choi et al., 2024), followed by rapid radiation that gave rise to a globally distributed lineage of morphologically diverse, complex macroalgae (Silberfeld et al., 2010; Starko et al., 2019; Bringloe et al., 2020).

While kelps and other seaweeds have been consumed by humans since the Mesolithic era (Buckley et al., 2023), kelp aquaculture development lagged compared to terrestrial agriculture until the 20th century (Hwang et al., 2019). Large scale kelp farming initially started in Japan, Korea, and China in the 1950s–1970s (Tseng and Fei, 1987), and Asia currently accounts for 97% of the $6 billion global kelp market (FAO, 2023; 2024). The predominant kelp species farmed are *Saccharina japonica* ($4.6 billion) and *Undaria pinnatifida* ($1.9 billion) (Cai et al., 2021), with kelp aquaculture directly supporting a range of industries, from food to pharmaceuticals (Kim et al., 2017). As the industry expanded, kelp breeding programs were formed to address low quality seed, increasing biomass, disease resistance, and trait consistency using phenotypic selection (Hwang et al., 2019; Hu et al., 2023). In agriculture, emerging genetic resources, such as reference genomes and sequence information for breeding panels of plants, have accelerated genomics guided breeding programs to develop more productive and resilient cultivars (Heffner et al., 2009). Genomics can also accelerate kelp breeding programs (Budhlakoti et al., 2022; Song et al., 2023), if the proper genetic resources, such as reference genomes and breeding populations, are developed (DeWeese and Osborne, 2021).

The haplodiplontic life cycle of kelp is ideal for genomics-based breeding, as haploid gametophytes can be vegetatively propagated in culture (Dring and Lüning, 1975; Huang et al., 2022; 2023). These gametophyte cultures can then serve as kelp breeding germplasm, with scalable production of monoclonal gametophyte cultures producing ample material for crossing or sequencing experiments (Hoffmann and Santelices, 1991). Sequencing data can then be aligned to reference genomes, producing variant information that can be used with phenotypic data to produce genomic selection models (Moose and Mumm, 2008). These models produce genome estimated breeding values (GEBVs) (Meuwissen et al., 2001), which can then be used to predict the phenotypes, such as biomass, of potential crosses in the sequenced germplasm (Desta and Ortiz, 2014; Huang et al., 2022). Sequenced germplasm collections, along with the vegetative propagation of haploid gametophytes, compose an incredibly powerful tool for breeding programs for kelp (The Aquaculture Genomics, Genetics and Breeding Workshop et al., 2017; Wade et al., 2020).

The brown macroalga *Saccharina latissima* (sugar kelp, Laminariales) (Lane et al., 2006) is an ecologically and economically significant species found in the Pacific, Atlantic, and Arctic Oceans (Diehl et al., 2024). *S. latissima* is a sister species to the commercially cultivated Japanese sugar kelp *S. japonica*, with a similar morphology of an undivided single blade (Redmond et al., 2014), ideal for kelp farming (Peteiro and Freire, 2011). *S. latissima* is farmed in both northern Europe and North America, and is the most farmed kelp in the United States, accounting for a predominant share of the current >$300 million US kelp industry (Kim et al., 2019a; Heidkamp et al., 2022; Brayden and Coleman, 2023; Stekoll et al., 2024). Kelp mariculture is a rapidly developing sector in the United States and demands for fundamental research into cultivable kelp species have engendered significant investment (e.g., ARPA-E MARINER programs) in projects to increase the productivity of kelp farms (Li et al., 2022).

While population genetic studies of sugar kelp in both the United States and Europe have begun to provide some of the resources necessary for breeding programs (Breton et al., 2018; Mao et al., 2020; Huang et al., 2022), an annotated reference genome is foundational for genomic selection technologies. In fact, early results from a *S. latissima* genomic selection breeding program based on the reference genome described here produced cultivars that doubled biomass yield compared with non-selected kelps (Huang et al., 2023). The recently published European *S. latissima* genome (Denoeud et al., 2024) represents an important milestone in research and development for the sugar kelp aquaculture industry in Europe and for expanded brown algal genetic analyses through the Phaeoexplorer genome database. In the United States, a reference for North American *S. latissima* provides opportunities to further refine breeding models on local sugar kelp populations for the expanding kelp industry (Brakel et al., 2021), as well as to aid in kelp forest conservation and restoration efforts (Coleman and Glasby, 2024; Bemmels et al., 2025). We present the scaffolded and annotated nuclear and organelle genome assemblies of North American sugar kelp (*S. latissima*), key genomic resources for population genetic studies, conservation, and modern genomic breeding of sugar kelp in the United States.

## 2 Materials and methods

### 2.1 Sample collection and nucleotide extraction

Reproductive sorus tissue from a wild population of *S. latissima* sporophytes was sampled from Black Ledge, Groton, Connecticut, US (41°31′N, 72°07′W, 26 June 2014). Following induced sporulation, individual gametophytes were isolated to establish monoclonal gametophyte cultures in a laboratory setting, as outlined by Redmond et al. (2014) and Alsuwaiyan et al. (2019). One female *S. latissima* gametophyte (var. SL-CT1-FG3) was selected for long-read sequencing for reference genome assembly and cultured in Erlenmeyer flasks under red light with a 12:12 h light:dark photoperiod at 10°C to inhibit reproduction and promote growth and mitotic division (Redmond et al., 2014; Augyte et al., 2018) for genomic DNA extraction. Augyte et al. (2018) at the Marine Biotechnology Laboratory at the University of Connecticut extracted DNA from a 24 mg (fresh) female gametophyte culture of *S. latissima* (var. SL-CT1-FG3) using a modified protocol of the NucleoSpin Plant II Maxi Kit (Macherey-Nagel, Düren, Germany; cat # 740609). Gametophyte biomass was spun down in 1.5-mL tubes in an Eppendorf 5424 microcentrifuge (21,000 rcf, 2 min). Sealed tubes of gametophyte material were frozen in liquid nitrogen for 20 s before being ground with a plastic pestle for 30 s. Ground samples were extracted with CTAB buffer using repeated wash steps (Doyle and Doyle, 1990; Augyte et al., 2018).

We collected samples from Saccharina latissima sporophytes cultivated and harvested by University of Connecticut and GreenWave from a farm site in Branford, Connecticut, USA (41°15′13″N, 72°46′5″W, May 2020). Sporophytes were replicates of a single cross from *S. latissima* gametophyte collections (Augyte et al., 2018; Mao et al., 2020): a female gametophyte (var. LIS-F1-3) originating from Southern New England, and a male gametophyte (var. SL-CT1-MG2) cultured from the spore release that also generated SL-CT1-FG3. We flash-froze the samples in liquid nitrogen immediately after collection and subsequently stored at −80°C for less than 1 month before being sent on dry ice to Cornell University for RNA extraction. Sporophyte samples were ground in liquid nitrogen and RNA was extracted using the Quick-RNA Microprep Kit (Zymo Research, Irvine, CA, USA; cat #R1052), yielding 8.31 µg total RNA (average 1.66 µg/sample). An additional RNA extraction was performed at the HudsonAlpha Institute (Huntsville, Alabama, USA) from SL-CT1-FG3 gametophyte culture using the RNeasy Micro Kit (Qiagen Inc., Valencia, CA, USA; cat # 74004).

### 2.2 DNA and RNA sequencing

Extracted DNA from the *S. latissima* female gametophyte (var. SL-CT1-FG3) was sent to the HudsonAlpha Institute for whole genome sequencing using a whole genome shotgun sequencing strategy and standard sequencing protocols. Sequencing reads were collected using two platforms: Illumina reads were sequenced using the Illumina NovaSeq 6000 platform, and the PacBio reads were sequenced using the Sequel II platform. One 400bp insert 2 × 250 Illumina fragment library (66.03x) was sequenced along with one 2 × 150 Dovetail Hi-C library (145.33x) (Supplementary Table S1). Prior to use, the Illumina fragment reads were screened for phix contamination. Reads composed of >95% simple sequence were removed. Illumina reads <50bp after trimming for adapter and quality (q < 20) were removed. The final Illumina read set consisted of 282,564,006 reads for a total of 66.03x of high-quality bases. PacBio sequencing yielded 111.14 Gb of total raw sequence, representing 180.56x genomic coverage (Supplementary Table S2).

RNA extracted from five *S. latissima* sporophyte samples was sent to the HudsonAlpha Institute for library preparation and sequencing using standard protocols. All RNA libraries were prepared using TruSeq Stranded mRNA Library Prep (96 samples) (Illumina, San Diego, CA, USA; cat # 20020595) and indexed with IDT for Illumina TruSeq RNA UD Indexes v2 (96 indexes) (Illumina, San Diego, CA, USA; cat # 20040871) according to manufacturer instructions. cDNA was sequenced on the Illumina NovaSeq 6000 platform and generated a total of 287 million reads, with an average of 96 million reads per sample.

### 2.3 Nuclear genome assembly and decontamination

The initial assembly version 0 was generated by assembling 11,430,834 PacBio CCS reads of the female *S. latissima* gametophyte SL-CT1-FG3 using hifiasm v0.7 (Cheng et al., 2021) and subsequently polished using RACON v1.4 (Vaser et al., 2017). This produced an initial assembly (SL-CT1-FG3 v0) consisting of 4,854 contigs, with a contig N50 of 863.2 Kb, and a total assembly size of 925.8 Mb (Supplementary Table S3).

Hi-C sequencing of SL-CT1-FG3 yielded 626,664,456 2 × 150 Hi-C Illumina reads, an estimated 145.33x coverage. The reads were aligned to the SL-CT1-FG3 v0 assembly using BWA-MEM v0.7.17 (Li, 2013). Paired-end reads were mapped independently (as single-ends) due to the nature of the Hi-C pair, which captures conformation via proximity-ligated fragments. A small fraction of single-end mapped reads will contain a ligation junction, an indicator that they are chimeric because they do not originate from a contiguous piece of DNA. In these cases, only the 5′-side was retained, as the 3′-end generally originates from the same contiguous DNA as the 5′-side of the mated read. The resulting single end alignments were combined into a BAM file containing the paired, chimera-filtered Hi-C read alignments. The 3D-DNA (Dudchenko et al., 2017) suite of internal tools was used to generate a contact map using the resultant BAM file, and the contact map was visualized using Juicebox (Durand et al., 2016). Chromosome-scale scaffolding was attempted using 3D-DNA, but high levels of contamination in the v0 genome initially prevented meaningful scaffolding.

The assembled contigs were screened against bacterial proteins, organelle sequences, and the NCBI non-redundant protein sequence database (NR) (Sayers et al., 2024) and removed if found to be a contaminant according to standard practice (e.g., (Bennetzen et al., 2012; Motamayor et al., 2013; Bartholomé et al., 2015). Contigs were classified into bins depending on sequence content. Contamination was identified using BLASTn (Camacho et al., 2009) against the NCBI non-redundant nucleotide database (NT) (Sayers et al., 2024) and BLASTx using a set of known microbial proteins. Additional contigs were classified in the version 1 release as contaminants (2,863 contigs, 283.9 Mb), chloroplast (181 contigs, 11.0 Mb), prokaryote (14 contigs, 10.6 Mb), redundant (>95% masked with 24mers that occur more than 2 times in all contigs) (233 contigs, 6.2 Mb), repetitive (>95% masked with 24mers that occur more than 4 times in contigs greater than the contig N50) (24 contigs, 1.8 Mb), and unanchored rDNA (Ding et al., 2022) (3 contigs, 165.1 Kb).

### 2.4 Hi-C scaffolding and polishing

With contaminant contigs removed, we attempted to scaffold contigs together using the contact information from 3D-DNA (Dudchenko et al., 2017) and scaffold graph construction, as described by Ghurye et al. (2019). In brief, a graph was formed between all contigs in which graph edge weights, *w(u, v)*, between any two contigs *u* and *v* were computed by dividing the number of counts, *N(u, v)*, by the total number of cut sites, *C*, in both *u* and *v*: (Ghurye et al., 2019)

$$w\left(u,v\right)=\frac{N\left(u,v\right)}{C\left(u\right)+C\left(v\right)}$$

We then computed a normalized Best Buddy Weight, *BBW(u, v)*, as the weight, *w(u, v)*, divided by the maximal weight of any edge incident upon contigs *u* or *v*, excluding the *(u, v)* edge itself (Ghurye et al., 2019). All *BBW(u, v)* values >1 were retained, and a reverse Dijkstra’s algorithm (highest weight graph) (Dijkstra, 1959) was then utilized to generate the contig order and orientation for the join file. Using this algorithm, a total of 333 joins were made to form an additional 218 scaffolded contig sets. Each join was padded with an unsized gap of 10,000 Ns.

SNP and INDEL errors in the consensus were corrected with 282,564,006 Illumina fragment 2 × 250 reads (66.03x coverage) by aligning the reads using BWA-MEM (Li, 2013) and identifying SNPs and INDELs with GATK3 UnifiedGenotyper (Van der Auwera and O’Connor, 2020). A total of 502 SNPs and 20,723 INDELs were corrected in the release. The final version 1 release contained 612.21 Mb of sequence, consisting of 218 scaffolds and 1,513 contigs, with a contig N50 of 971.7 Kb (Supplementary Table S4).

### 2.5 Gene annotation

The JGI Annotation Pipeline (Grigoriev et al., 2006) was used to annotate the *S. latissima* nuclear genome assembly. The pipeline automatedly predicts, filters, and functionally annotates gene models as described by Grigoriev et al. (2006). Briefly: repeats in the genome assembly were masked with RepeatMasker (Smit et al., 2004), RepBase (Jurka et al., 2005), and RepeatScout (Price et al., 2005). The masked assembly was then used to predict protein-coding gene models with *ab initio*, homology, and transcriptomic modeling methods. *Ab initio* gene predictions were performed with Fgenesh (Salamov and Solovyev, 2000) and GeneMark (Ter-Hovhannisyan et al., 2008). Homology was assessed by BLASTx (Camacho et al., 2009) of the assembly against NCBI NR (Sayers et al., 2024). Resulting alignments were used to seed Fgenesh+ (Salamov and Solovyev, 2000) and Genewise (Birney et al., 2004) for homology-based gene prediction. A transcriptome assembly was generated for *S. latissima* with Illumina RNAseq reads with Trinity v2.11.0 (Grabherr et al., 2011), and RNA reads were mapped back to the genome assembly with HISAT2 (Kim et al., 2019b). With these inputs, the transcriptome-based programs Fgenesh (Salamov and Solovyev, 2000) and combest (Zhou et al., 2015) were used to generate gene models. A total of 41,561 gene models predicted across all methods (all models) were filtered based on protein homology and transcriptome evidence to a single representative model at each genomic locus (filtered models), generating a set of 24,790 gene models; for a detailed description of JGI model filtration, refer to Grigoriev et al. (2006). Protein sequences predicted from the set of filtered gene models were functionally annotated. Proteins were classified using SignalP v3 (Nielsen et al., 1997) for signal sequences, TMHMM (Melén et al., 2003) for transmembrane domains, and InterproScan (Quevillon et al., 2005) for functional domains. BLASTp (Camacho et al., 2009) alignments of proteins against NCBI NR (Sayers et al., 2024), Swiss-Prot (UniProt Consortium, 2012), KEGG (Kanehisa, 2004), and KOG (Koonin et al., 2004) databases to further informed functional interpretation of predicted proteins. The version 1 *S. latissima* genome assembly, associated annotations, and metadata are hosted on the JGI PhycoCosm (Grigoriev et al., 2021) comparative algal genome portal (https://phycocosm.jgi.doe.gov/SlaSLCT1FG3_1).

### 2.6 Comparative genomic analyses

Gene content was scored for the analyzed brown macroalgal genomes using BUSCO v5.7.1 (Manni et al., 2021) in genome mode with gene predictor set to Augustus v3.5.0 (Stanke et al., 2008) against the ortholog databases for Eukaryota (eukaryota_odb10) and Stramenopiles (stramenopiles_odb10). QUAST-LG v5.2.0 (Mikheenko et al., 2018) was used to compute relevant assembly quality metrics for each. Synteny of our v1 *S. latissima* assembly to related species was investigated by aligning to published genomes of *S. japonica*, *Macrocystis pyrifera*, *U. pinnatifida*, and *Ectocarpus* sp. using the Progressive Cactus v2.6.7 pipeline (Armstrong et al., 2020). The phylogenetic tree used to seed the Cactus alignment was pruned using tidytree v0.4.6 (Yu, 2022) and treeio v1.30.0 (Wang et al., 2020) from the Starko et al. (2019) kelp phylogeny constructed from plastid, mitochondrial, and ribosomal genes.

Blocks of synteny were extracted from the five-species hierarchical whole genome alignment (HAL) format (Hickey et al., 2013) into the BLAT-defined PSL format (Kent, 2002) using halSynteny v2.2 (Krasheninnikova et al., 2020). We applied a method put forward by Nosil et al. (2023) to establish one-to-one homology between chromosomes of related species using synteny blocks derived from pairwise Cactus alignments. To appropriately map our *S. latissima* scaffolds onto longer reference chromosomes, we modified the method to a many-to-one approach, allowing multiple *S. latissima* scaffolds to align to a single chromosome. For each alignment of a *S. latissima* scaffold to a reference species chromosome, lengths of syntenic blocks (“matches” in PSL format (Kent, 2002)) were summed. Best scaffold-chromosome pairs were identified with respect to each *S. latissima* scaffold, hereafter referred to as a “maximal syntenic match”, by calculating the maximum summed exact match per scaffold amongst the aligned chromosomes. FASTA genome assembly files for each of the five species, and a whole genome alignment file converted from HAL to MAF using hal2maf v2.2 (Hickey et al., 2013), were given as input to Ragout v2.3 (Kolmogorov et al., 2018), a reference-assisted scaffolding tool used to improve assembly contiguity.

Heatmaps representing synteny between reference chromosomes of each species versus *S. latissima* scaffolds were generated using ggplot2 v3.5.1 (Wickham, 2016) in R v4.4.0 (R Core Team, 2024). Ordering of our genome v1 assembly scaffolds onto synteny-constructed pseudochromosomes was rendered into genetic map representation using ggplot2 v3.5.1 (Wickham, 2016) in R v4.4.0 (R Core Team, 2024). Scripts used to perform these analyses and generate figures are hosted in a public GitHub repository (https://github.com/kdews/s-latissima-genome).

### 2.7 NCBI decontamination

The v1 assembly was screened for vector using the NCBI UniVec database (https://www.ncbi.nlm.nih.gov/tools/vecscreen/univec) using the standard command blastall -p blastn -d Saccharina_latissima.mainGenome.fasta -i UniVec -q −5 -G 3 -E 3 -F ″m D” -e 700 -Y 1.75e12 -m 8 (Camacho et al., 2009). A total of 43 vector hits were identified with≥95% identity and≥31bp in length. Contaminants were identified using the contaminant screener FCS-GX (Astashyn et al., 2024). A total of 90 contaminated regions were identified (63 TRIM, 21 FIX, 6 EXCLUDE). All identified vector and contaminant regions were removed from the sequence. If a vector/contaminant region fell within a scaffold, then the region was replaced with the same number of N’s. If the vector/contaminant region fell on the front/end of a scaffold, the bases were eliminated, and the annotation GFF file was translated appropriately. These changes resulted in a net loss of 53 scaffolds, 4,498,393 bp, and a total of 209 annotated genes.

### 2.8 Organelle genome assemblies

Organelle genomes from the same female *S. latissima* gametophyte (SL-CT1-FG3) were also assembled using the PacBio reads generated for the nuclear genome assembly. Reference organelle genomes for *S. latissima* have been published (Wang et al., 2016; Fan et al., 2020b), but these genomes do not have corresponding nuclear genomes from the same individual. Gene transfers between organelle and nuclear genomes are more easily identified when using genomes sourced from the same individual (Cui et al., 2021). To identify organelle reads, raw PacBio reads were aligned to the *S. latissima* mitochondrial (Wang et al., 2016) and chloroplast (Fan et al., 2020b) genomes using minimap2 standard settings (Li, 2018). Read IDs that aligned to the organelle genomes were extracted using samtools (Li et al., 2009), and then corresponding PacBio reads that aligned to the respective organelle genomes were subset using seqtk subseq (Li, 2012). Organelle genomes were then assembled using the assembler flye v2.9.2-b1786 (Kolmogorov et al., 2019), with the--pacbio-raw flag and genome size estimates based on previously published sugar kelp chloroplast (Fan et al., 2020b) and mitochondrial (Wang et al., 2016) genomes. We used GeSeq2 (Tillich et al., 2017) to annotate and compare our sugar kelp organelle genomes versus available published reference *S. latissima* organelle genomes (Wang et al., 2016; Fan et al., 2020b), using genome annotations of *U. pinnatifida* mitochondria (Li et al., 2015) and chloroplast (Zhang Y. et al., 2016) as outgroups. A collection of scripts used in this analysis have been placed in a public GitHub repository (https://github.com/kdews/s-latissima-organelles).

## 3 Results

### 3.1 Nuclear genome assembly

#### 3.1.1 General statistics

**FIGURE 1:**
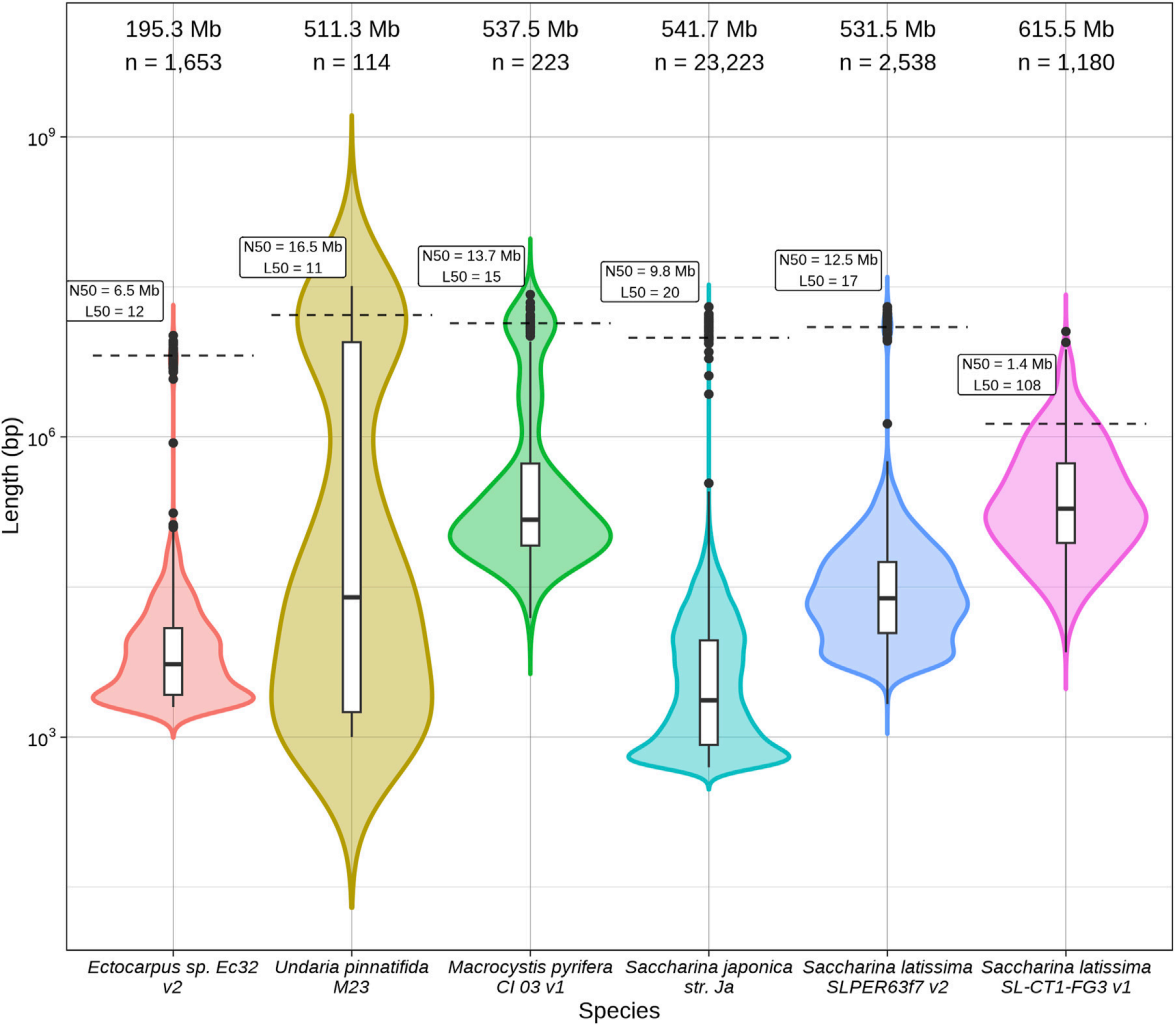
Log_10_-scaled size distribution for six brown algae genome assembly contigs and scaffolds: *Ectocarpus* sp. Ec32 v2 (Cormier et al., 2017), *Undaria pinnatifida* M23 (Shan et al., 2020), *Macrocystis pyrifera* CI_03 v1 (Diesel et al., 2023), *Saccharina japonica* str. Ja (Fan et al., 2020a), European *Saccharina latissima* SLPER63f7 v2 (Denoeud et al., 2024) and North American *Saccharina latissima* SL-CT1-FG3 v1 (this publication).

Long-read sequencing of an individual *S. latissima* gametophyte (var. SL-CT1-FG3) yielded 111 Gb of PacBio HiFi reads, estimated to represent ∼180x genomic coverage. Our initial *de novo* assembly (v0) was 925.8 Mb and consisted of 4,854 contigs (Supplementary Table S3). Following contaminant filtering, a total of 3,341 contigs (283.9 Mb) were removed. Hi-C scaffolding joined 333 contigs from the v0 genome into 218 scaffolds in the v1 genome. The final *S*. *latissima* genome (v1) described here contains a total of 218 scaffolds and 1,513 contigs, with a genome size of 615.5 Mb (0.5% gaps) and scaffold N50 of 1.35 Mb (Figure 1; Table 1). Gene annotation with the JGI annotation pipeline (Grigoriev et al., 2006) yielded 24,790 filtered gene models and 25,012 functionally annotated protein sequences. All annotations are available on the JGI PhycoCosm portal (Grigoriev et al., 2021) for our *S. latissima* v1 genome (https://phycocosm.jgi.doe.gov/SlaSLCT1FG3_1).

**TABLE 1:** Comparison of *Saccharina latissima* nuclear genome assembly statistics to those of related brown macroalgal species. Number of genes reported reflects conservatively curated gene models reported for each specific genome assembly. *Excludes artificial chromosomes.

	*Ectocarpus* sp. Ec32	*Undaria pinnatifida*	*Macrocystis pyrifera*	*Saccharina japonica*	*Saccharina latissima* (European)	*Saccharina latissima* (American)
Genome size (Mb)	196.8	511.3	537.5	548.5	531.4	615.5
Genome size in chromosomes	91%	98%	92%	65%	79%	46% (scaffolds)
Chromosomes*	28	30	35	31	31	*est. 32*
Scaffolds*	28	114	35	31	2,536	218
Contigs	12,767	618	921	37,788	4,592	1,513
Largest scaffold (Mb)*	10.32	32.30	26.51	19.97	19.99	11.32
Scaffold N50 (Mb)*	6.53	16.51	13.67	12.42	12.52	1.35
Contig N50 (Kb)	32	1,800	1,000	44	247	971
Percent gaps	2.602%	0.049%	0.013%	1.733%	0.040%	0.541%
GC content	53.59%	50.14%	50.37%	49.66%	49.78%	49.84%
Genes	18,369	12,499	25,919	50,098	18,169	25,012
Complete BUSCOs Stramenopiles	95.0%	92.0%	94.0%	87.0%	88.0%	86.0%
Complete BUSCOs Eukaryota	69.0%	70.6%	69.8%	57.7%	60.8%	59.2%
Genome coverage	121x	120x	100x	178x	–	185x
Citation	Cormier et al. (2017)	Shan et al. (2020)	Diesel et al. (2023)	Fan, et al. (2020a)	Denoeud et al. (2024)	This publication

To evaluate the quality and completeness of the North American *S. latissima* genome assembly reported here, we compared to the genomes of four related brown algae: the model brown alga *Ectocarpus* sp. (Cormier et al., 2017), giant kelp *M. pyrifera* (Diesel et al., 2023), and the widely cultivated kelps *wakame U. pinnatifida* (Shan et al., 2020) and Japanese sugar kelp *S. japonica* (Fan et al., 2020a) (Figure 1; Table 1). Following the recent publication of the Phaeoexplorer brown algal genome database (Denoeud et al., 2024), the European *S*. *latissima* nuclear assembly was also included for comparison on summary statistics (Figure 1; Table 1). Three early genome size predictions for *S. latissima* used standard methods of staining and flow cytometry to estimate a genome size of 588–720 Mb (Phillips et al., 2011). These genome size estimates agree with our v1 assembly size of 615.5 Mb. The sequenced length and GC content of this *S. latissima* genome assembly is generally comparable to the other two *Saccharina* genomes; our North American *S. latissima* assembly contains ∼84 Mb more sequence than the European assembly (Table 1).

#### 3.1.2 Conserved gene content

**FIGURE 2:**
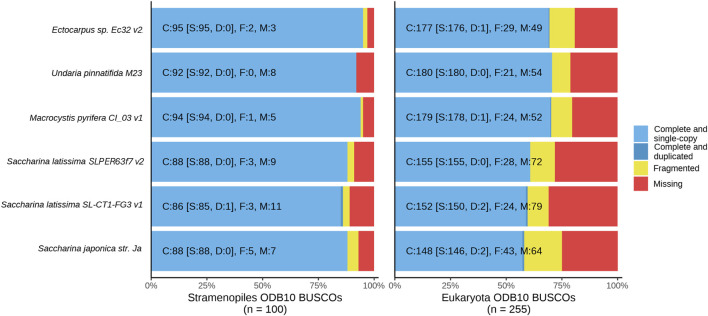
BUSCO scoring using the Stramenopiles and Eukaryota ortholog databases (odb10) shows relative counts of complete (blue), fragmented (yellow), and missing (red) orthologs in each compared brown algae assembly, *Ectocarpus* sp. Ec32 v2 (Cormier et al., 2017), *Undaria pinnatifida* M23 (Shan et al., 2020), *Macrocystis pyrifera* CI_03 v1 (Diesel et al., 2023), *Saccharina japonica* str. Ja (Fan et al., 2020a), European *Saccharina latissima* SLPER63f7 v2 (Denoeud et al., 2024) and North American *Saccharina latissima* SL-CT1-FG3 v1 (this publication).

Gene content was evaluated with BUSCO, which evaluates a given assembly against the set of single-copy, highly conserved orthologous genes predicted to be present in a specific clade (Manni et al., 2021). Detection of conserved gene orthologs in *de novo* assemblies infers genome completeness, especially when comparing single-copy orthologs amongst species within a monophyletic clade (Waterhouse et al., 2011). We benchmarked the six compared genomes against two relevant clades: Eukaryota, which provides a general metric for comparison across all conserved Eukaryota genes, and Stramenopiles genes, the monophyletic clade containing brown algae. The percentage of complete BUSCOs (comprising single copy and duplicate orthologs) detected in an assembly can be used as a proxy for genome completeness and to detect artificial duplications resulting from *de novo* assembly. Benchmarked against Eukaryota, our North American *S. latissima* assembly (59.2% complete BUSCOs) scores closely to *S. japonica* (57.7%) and the European *S. latissima* assembly (60.8%); similarly, against Stramenopiles this assembly scored 86%, compared to 88% in *S. japonica* and the European *S. latissima* (Table 1; Figure 2). Of the 100 BUSCOs in Stramenopiles, our genome contains 86 complete BUSCOs (86%), with 85 single copy and 1 duplicated, and the remainder fragmented (3) and missing (11) (Figure 2). Incomplete BUSCOs in the *S. latissima* genome could be attributed to lower contiguity, which would be further exacerbated by the *Laminariales* gene structure that often features long intronic regions. Long genes split between unassembled regions could be fragmented beyond the threshold of local alignment detection.

### 3.2 Synteny analysis

#### 3.2.1 Interspecies whole genome alignment

**FIGURE 3:**
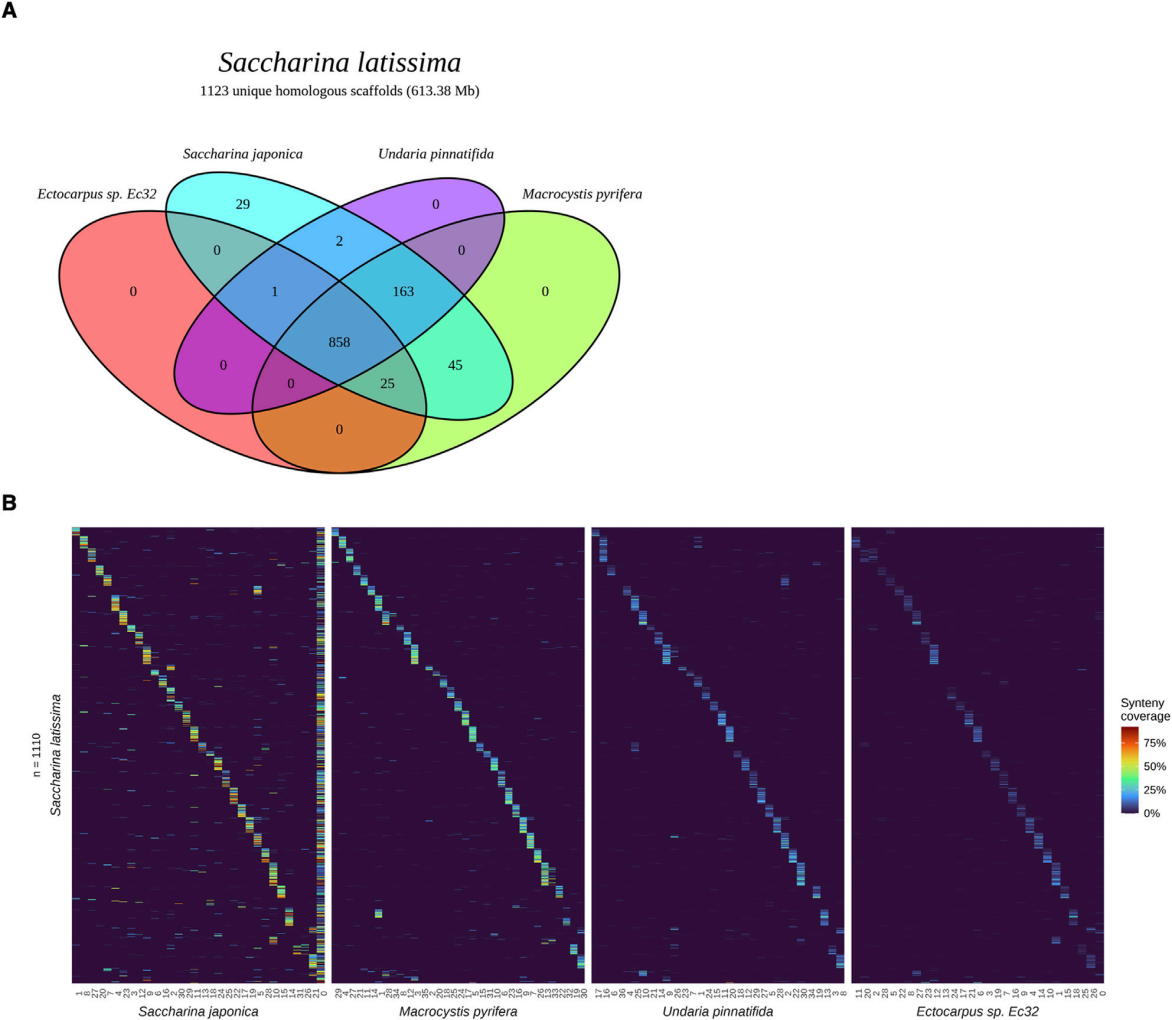
**(A)** Venn diagram highlights intersects between sets of homologous *Saccharina latissima* v1 assembly scaffolds that uniquely mapped to the genomes of four related species: *Ectocarpus* sp. Ec32, *Saccharina japonica*, *Undaria pinnatifida*, and *Macrocystis pyrifera*. **(B)** Heatmap shows the maximal syntenic match of 1,110 *S. latissima* scaffolds and contigs to chromosomes of the same four related brown algal species.

To capture canonical genomic rearrangements between related species, we performed hierarchal whole multi-genome alignments (Armstrong et al., 2020) of our *S. latissima* assembly against the genomes of four related brown macroalgal species, which formed the basis of our homology analysis. Sequence homology to at least one of the compared genomes was detected in 99% of our assembly across 1123 scaffolds and contigs (613.38 Mb). On average, exact matches spanned 15% of each scaffold in *S. latissima*. A core set of 858 scaffolds and contigs (525.43 Mb) in the *S. latissima* genome aligned the genome assemblies of each of the four compared brown algal species (Figure 3A).

As expected, *S. latissima* had the highest total exact matches to *S. japonica* of all species, both genome-wide (181.89 Mb) and averaged per chromosome (5.68 Mb) (Supplementary Table S5). Overall, summed exact matches between our v1 *S. latissima* assembly and each compared assembly increases with respective species relatedness to *S. latissima*, a trend that holds both genome-wide and per chromosome (Supplementary Figure S1B; Supplementary Table S5). Linear regression of *S. latissima* scaffold lengths versus their respective homologous reference chromosome lengths yielded slopes that closely correspond to genome size ratios, with the smallest reference, *Ectocarpus* sp. Ec32, reflecting a 3x shorter genome with a slope of 3.06 (Supplementary Figure S1A).

#### 3.2.2 Genome contiguity and chromosome number estimation

Despite high genomic sequencing coverage (185x) with long PacBio reads, as well as Hi-C sequencing to 145x coverage, our reported *S. latissima* v1 genome assembly could not be scaffolded to the level of chromosomes based on sequencing and contact information alone. Our v1 assembly contains 218 scaffolds, and the scaffold N50 of our v1 *S. latissima* assembly (1.35 Mb) is an order of magnitude smaller than similarly sized brown macroalgal genomes (Table 1). Chromosome number estimates in brown macroalgae are complicated due to sex-specific polyteny and population-specific ploidy variation in sugar kelp meristem sporophyte tissue (Müller et al., 2016; Goecke et al., 2022). Chromosome number predictions in *Saccharina* species vary depending upon which method is used: flow cytometry has estimated a genome ranging from 588 to 720 Mb with 62 chromosomes (Phillips et al., 2011), while microscopy has estimated 31 chromosomes (Liu et al., 2012; Liu et al., 2022). Recent brown macroalgal genome assemblies have predicted 28–34 chromosomes (Cormier et al., 2017; Fan et al., 2020a; Shan et al., 2020; Diesel et al., 2023). The European *S. latissima* genome (Denoeud et al., 2024) was able to scaffold 2,085 of 4,592 contigs into 31 pseudochromosomes using genetic linkage mapping in its v2 release (February 2025), which aligns with microscopy estimates for chromosome count in *Saccharina*.

We applied the results of our hierarchal whole-genome alignments of *S. latissima* to *Ectocarpus* sp. Ec32, *S. japonica*, *U. pinnatifida*, and *M. pyrifera* to identify chromosomes homologous to our v1 scaffolds. For each scaffold in the *S. latissima* v1 genome assembly that aligned, we calculated a maximal syntenic match to a single chromosome in each related genome (see Methods 3.6). Accounting for the sub-chromosomal length of our scaffolds, we assigned maximal syntenic matches with respect to *S. latissima*, with each scaffold matching only one chromosome, while chromosomes could be assigned multiple homologous *S. latissima* scaffolds (one-chromosome-to-many-scaffolds). With this schema, we visualized *S. latissima* scaffold alignments along the length of chromosomes in each of the four aligned genomes (Figure 3B).

**FIGURE 4:**
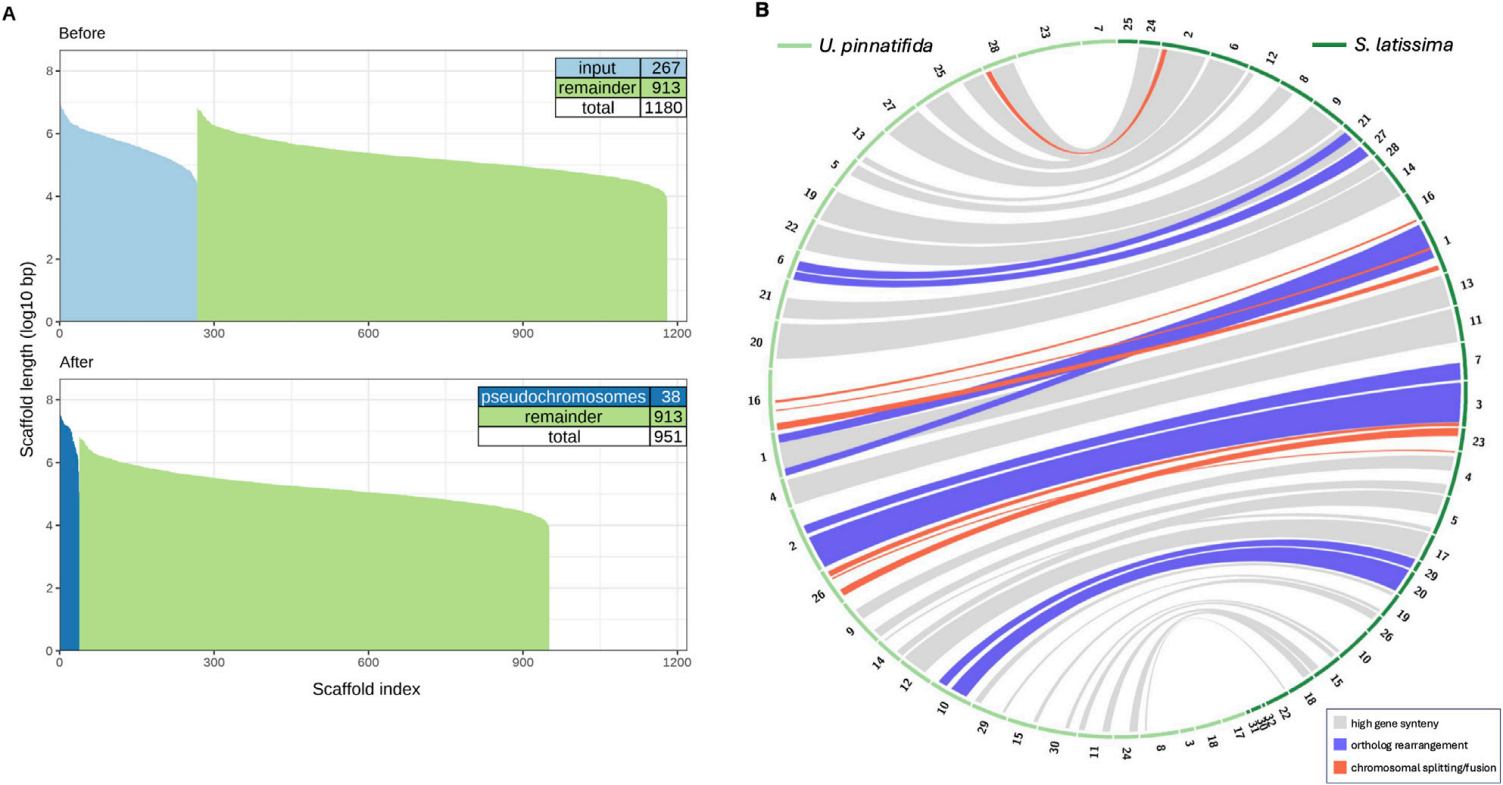
**(A)** Size distribution (log_10_ bp scale) of our v1 *Saccharina latissima* assembly before and after synteny-based re-scaffolding show 267 scaffolds (light blue) incorporated into 38 psuedochromosomes (dark blue). **(B)** Gene orthology between 32 *S. latissima* psuedochromosomes (dark green) and 38 *Undaria pinnatifida* chromosomes and contigs (light green) was mapped using 3 Mb windows containing 10 single-copy orthologs, with band colors denoting highest synteny (gray), ortholog rearrangement (red) and chromosomal splitting or fusion (purple).

We estimated chromosome number in *S. latissima* by leveraging syntenic information to order our assembly scaffolds into pseudochromosomes. We repeatedly re-scaffolded our v1 assembly under varying parameters (Kolmogorov et al., 2018) into 32–40 pseudochromosomes that incorporated 92–464 scaffolds and contigs (Supplementary Figures S2, 3), reflecting 40%–55% of genome sequence. Restricting our re-scaffolding to the 155 longest *S. latissima* scaffolds and allowing for chimeric assembly yielded 32 pseudochromosomes (Supplementary Figures S2D, S3D), the closest result to the predicted 31 chromosomes for *Saccharina*. This 32-pseudochromosome assembly was then used to map gene orthology between the genomes of *S. latissima* and *U. pinnatifida* (Shan et al., 2020) (Figure 4B).

### 3.3 Organelle genomes

Our assembled *S. latissima* chloroplast genome (130,613 bp) is almost the same length as the published chloroplast genome (130,619 bp) (Fan et al., 2020b) (Table 2; Supplementary Figure S4; Supplementary Figure S6B). Our assembled *S*. *latissima* mitochondrial genome is 37,510 bp and contains 39 genes. It is slightly smaller than the previously published mitochondrial genome (37,659 bp) (Wang et al., 2016), but contains an additional tRNA annotation (Table 2; Supplementary Figure S5; Supplementary Figure S6A). For each of the new organelle genomes reported here, a consensus sequence was generated through multiple separate assemblies with flye (Kolmogorov et al., 2019), a long-read assembler especially robust to sequencing errors and specialized to resolve repetitive regions. Long reads allowed for resolution of inverted repeat (IR) regions in the chloroplast genome that typically span ∼5.5 kb in brown macroalgae (Rana et al., 2019). The average sequencing read length (8,389 bp) also aided with assembly, as each read covers ∼22% and ∼6% of mitochondria and chloroplast genome lengths, respectively.

**TABLE 2:** Comparison of *Saccharina latissima* organelle genome versions on assembly and gene annotation statistics.

*Saccharina latissima* organelle genome (version)	Sequencer	Average read length (bp)	Size (bp)	Genes	tRNAs	rRNAs
Mitochondria (this publication)	PacBio Sequel II	8,389	37,510	39	19	3
Mitochondria (Wang et al., 2016)	Illumina HiSeq 2000	200	37,659	38	18	3
Chloroplast (this publication)	PacBio Sequel II	8,389	130, 613	138	27	3
Chloroplast (Fan et al., 2020b)	Illumina HiSeq 2000	200	130, 619	138	27	3

## 4 Discussion

### 4.1 Enhancing sugar kelp breeding with genomic tools

The decrease in sequencing costs has led to an increase in genome assemblies for non-model species including brown algae, which until recently have lacked genomic resources. Nuclear genomes are now becoming available for some Phaeophyta species, e.g., *Ectocarpus sp*. (Cock et al., 2010; Cormier et al., 2017), *S. japonica* (Ye et al., 2015; Liu et al., 2019; Fan et al., 2020a), *U. pinnatifida* (Shan et al., 2020; Graf et al., 2021), and *S. latissima* (Denoeud et al., 2024; this publication). This annotated and scaffolded genome of North American sugar kelp (*S. latissima*) represents a major step forward in kelp research and development in the United States. Our assembly provides a reference for future research into population genetics and gene expression in sugar kelp populations along the coast of the northeastern US and supports both marine conservation efforts and the nascent US kelp industry.

Many tools used in breeding rely on the availability of high-quality genomes and rigorous gene annotation. Demands on genome assembly coverage, accuracy, and annotation have increased as selective breeding methods have progressed (Varshney et al., 2014; International Wheat Genome Sequencing Consortium et al., 2018; Bayer et al., 2020). With sparse maps of genetic markers, marker-assisted selection (MAS) achieved modest improvements over phenotypic selection in the prediction of breeding values for crops and livestock (Dekkers and Hospital, 2002). Modern genomic selection (GS) models depend on high-density genotypic data to generate genomic estimated breeding values (GEBVs) (Meuwissen et al., 2001) that take genome-wide variation into account (Calus and Veerkamp, 2007; Heffner et al., 2009; Christensen and Lund, 2010). To accurately predict the performance of genetic crosses with these methods, chromosome rearrangements between them must be known (Nuzhdin et al., 2012). Additionally, genome engineering (e.g., CRISPR/Cas9 gene editing) relies on the construction of accurate genome assemblies for cultivated species to mitigate potential off-target genome modification (Guo et al., 2023). Here, we have assembled a genome for *S. latissima* of sufficient quality to assess genetic variation through a variety of markers (e.g., SNPs, INDELs, structural variants) useful for the progression of intraspecific breeding programs (Li et al., 2022), as well as possible crossbreeding with closely related species such as *S. japonica* (Zhang J. et al., 2016; 2018).

### 4.2 Improved organelle genome resources for sugar kelp

In addition to nuclear genomes of brown algae, publications of plastid and mitochondria genome assemblies are also mounting (Oudot-Le Secq et al., 2006; Chen et al., 2019; Rana et al., 2021). While genomics has greatly increased breeding efficiency in plants, animals, and kelp, most of these breeding programs rely strictly on nuclear genomes for establishing markers for selective breeding. However, integrating organelle genomes into breeding models may increase breeding efficiency (Kersten et al., 2016). By optimizing cytonuclear interactions, breeding efficiency for sugar kelp in the future can be accelerated (Colombo, 2019). As nuclear and organelle genomes can differ between individuals in the same species, pairing nuclear and cytoplasmic genomes from a single genotype can provide a reference for future breeding experiments. For *S. latissima*, short-read organelle genome assemblies have become available over the past decade (Wang et al., 2016; Fan et al., 2020b; Rana et al., 2021). Our use of long-read sequencing increases our confidence in the nuclear and organelle *S. latissima* genome assemblies described here to serve as a foundation for future sugar kelp scientific research and breeding.

### 4.3 Advancements in brown algal genome assembly and comparative genomics

The whole-genome hierarchal alignments conducted in this study produced syntenic data applicable to both our genome improvement and comparative genomic analyses, and future research into brown algae evolution, phylogenetics, and genomic breeding. High molecular weight DNA extraction is particularly challenging for macroalgal species (Snirc et al., 2010; Greco et al., 2014), which can pose a significant barrier to generating chromosome-level assemblies. In general, genome quality and completeness are assessed through gene annotation and evaluation of sequence contiguity. From a structural perspective, genome contiguity (i.e., N50, gaps/N content) informs the degree to which assembly and scaffolding methods have succeeded in reconstructing chromosomes from whole-genome shotgun sequencing.

In brown macroalgae, *Ectocarpus* sp. Ec32 v2 (Cormier et al., 2017), *U. pinnatifida* (Shan et al., 2020), and *M. pyrifera* (Diesel et al., 2023) represent the most highly scaffolded genome assemblies, with greater than 90% of sequence contained in chromosomal scaffolds. In contrast, although the *S. japonica* assembly (Fan et al., 2020a) has been mapped into chromosomes, almost 35% of the genome is not scaffolded. A similar pattern exists in the European *S. latissima* v2 assembly (Denoeud et al., 2024), in which >20% of the genome was not incorporated into pseudochromosomes. Our North American *S. latissima* v1 genome assembly contains 218 scaffolds that incorporate ∼45% of the genome by length. These results are despite high (>100x) genomic coverage with long reads and Hi-C-assisted assembly methods for all genomes compared in this study (Table 1). Though not conclusive, the relative difficulty of *de novo* chromosomal reconstruction in *Saccharina* genome assemblies could indicate physical traits or genome architecture specific to this genus that impede efforts to sequence and scaffold DNA, for example, elevated concentrations of polysaccharides and/or phenols or higher genomic repeat content in the genus *Saccharina* as compared to other kelps. To overcome obstacles to chromosome-level *de novo* assembly, we demonstrate the utility of syntenic information to estimate chromosome number and assist in scaffold placement along chromosomes in *S. latissima*.

Importantly, our comparative genomics analysis has identified syntenic regions and homologous chromosomes between five species of brown algae. Construction of the brown algal phylogeny has historically been complicated and contested, both in morphological and genetic studies, owing to a sparse brown algal fossil record, rapid diversification, and convergent evolution (Bolton, 2010; Silberfeld et al., 2010; Starko et al., 2019; Bringloe et al., 2020; Choi et al., 2024; Denoeud et al., 2024). Building on syntenic blocks we identified among brown algal genomes, phylogenomic analyses that evaluate whole genomes and investigate chromosome homology and evolution have the potential to resolve outstanding problems of placement within this clade radiation (Steenwyk and King, 2024). Comparative genomic analyses like those presented here help correlate chromosomes between species for more robust evolutionary analysis and genomic breeding prediction that leverage the increasing amount of high-quality, functionally annotated brown macroalgal genome assemblies.

## 5 Future directions

This scaffolded and annotated sugar kelp (*S. latissima*) genome is a fundamental resource for subsequent basic science research, genomic breeding, and conservation in sugar kelp. Already, this genome has served as the backbone of recent applied genomic advances of a sugar kelp selective breeding project, including a genomic selection model targeting higher yield (Umanzor et al., 2021; Li et al., 2022; Huang et al., 2023). Despite several years of work on the nuclear genome assembly for *S. latissima*, the reference can and should be further improved with the goal of a chromosome-level genome. As we have demonstrated, this and other high-quality brown macroalgae genomes allow for comparative genomics studies to examine the evolution of chromosome structure and gene content across brown algae. To investigate sugar kelp genetic diversity and evolution across important biogeographic regions such as Alaska, Gulf of Maine, and Europe, future work should look to assemble a sugar kelp pan-genome representing global genetic variation across known sugar kelp populations.

The importance of genomics to kelp domestication projects has been highlighted by other industry leaders (Goecke et al., 2022; Hu et al., 2023). Since sugar kelp is the most farmed kelp in the United States and Europe, publication of an annotated reference genome for North American sugar kelp alongside the existing European sugar kelp genome (Denoeud et al., 2024) will advance breeding opportunities for the kelp farming industry worldwide. The knowledge presented here is crucial to genomic selection for superior traits, such as high yield, disease resistance, and stress tolerance, to bring improved sugar kelp cultivars to United States markets.

## Data Availability

The datasets presented in this study can be found in online repositories. The names of the repository/repositories and accession number(s) can be found below: PRJNA1156187 (https://www.ncbi.nlm. nih.gov) and SlaSLCT1FG3_1 (https://phycocosm.jgi.doe.gov/ SlaSLCT1FG3_1).
