# Supplementary material for: Scaffolded and annotated nuclear and organelle genomes of the North American brown alga Saccharina latissima
Source: Front Genet. 2025 May 14;16:1494480. doi: 10.3389/fgene.2025.1494480 (PMC12116465; doi:10.3389/fgene.2025.1494480)
Supplement: Supplementary file 1 [file Supplementaryfile1.docx]

Supplementary Material

## Supplementary Figures


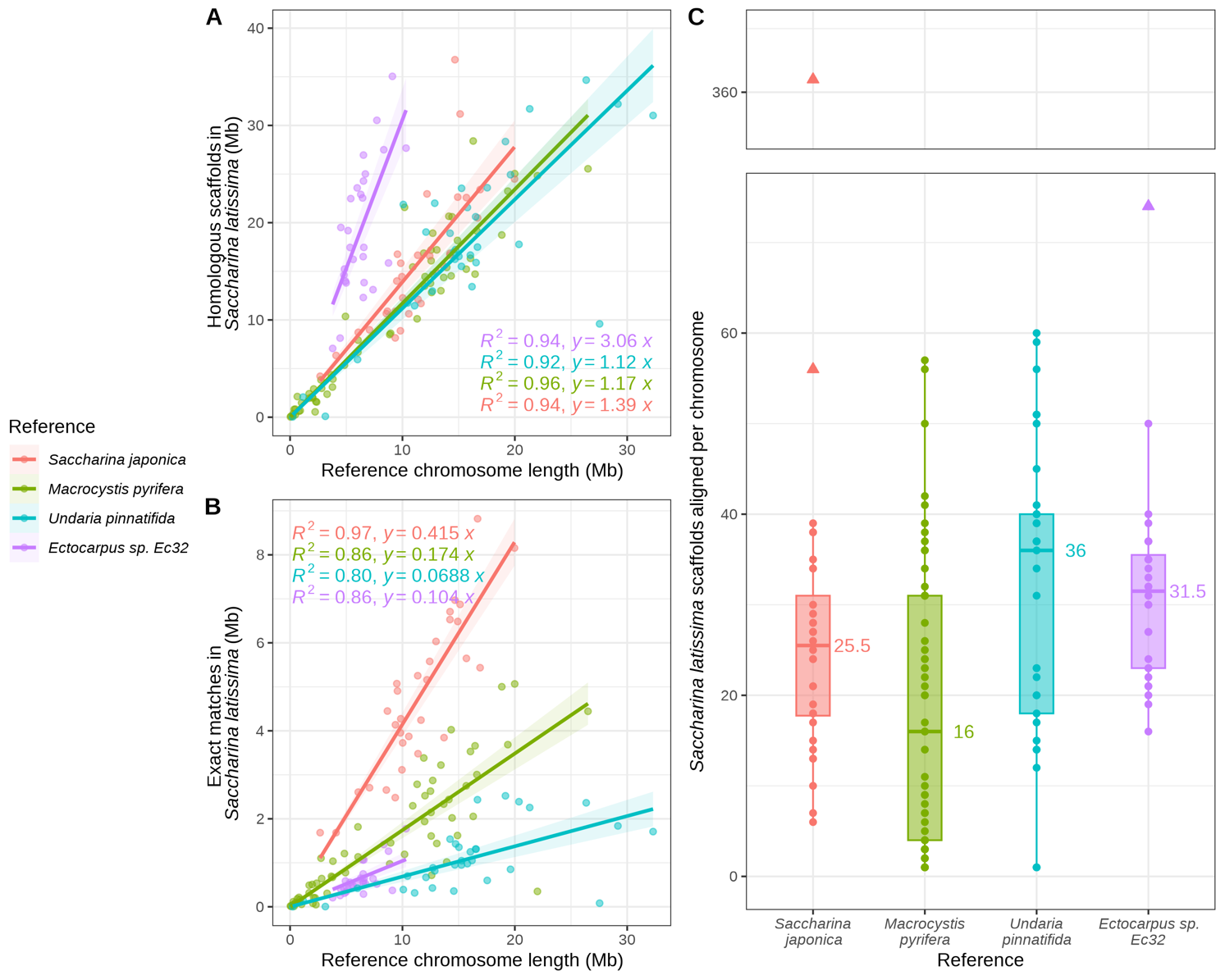


**Supplementary Figure 1.** Regression fits of total lengths **(A)** and alignment lengths **(B)** of *Saccharina latissima* scaffolds and contigs versus homologs in *Saccharina japonica* (red), *Macrocystis pyrifera* (green), *Undaria pinnatifida* (blue), and *Ectocarpus* sp. Ec32 (purple). Excludes artificial chromosomes. **(C)** Distributions of n *S. latissima* scaffolds aligned to each reference chromosome. Outliers are marked with triangles.


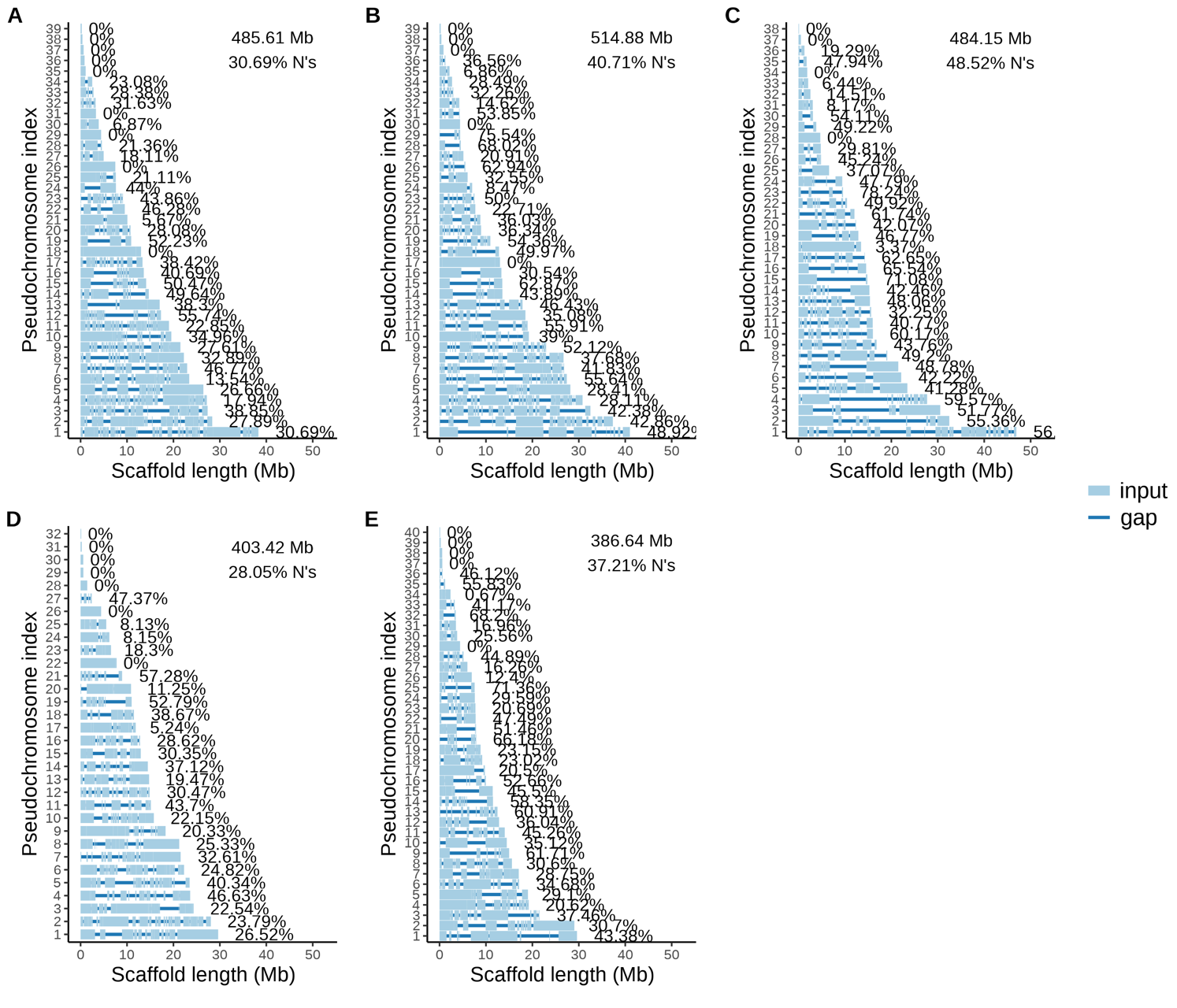


**Supplementary Figure 2.** Arrangement of v1 assembly scaffolds and contigs (light blue “input” bars) onto pseudochromosomes. Each plot shows the result of re-scaffolding the v1 *Saccharina latissima* assembly with varying parameters given to Ragout: (A) no size filtering, chimeric assembly allowed, (B) no size filtering, unbroken input scaffolds, (C) no size filtering, unbroken input scaffolds, forced HAL instead of MAF, (D) short contigs filtered out of comparison genomes, chimeric assembly allowed, (E) short contigs filtered out of comparison genomes, unbroken input scaffolds.


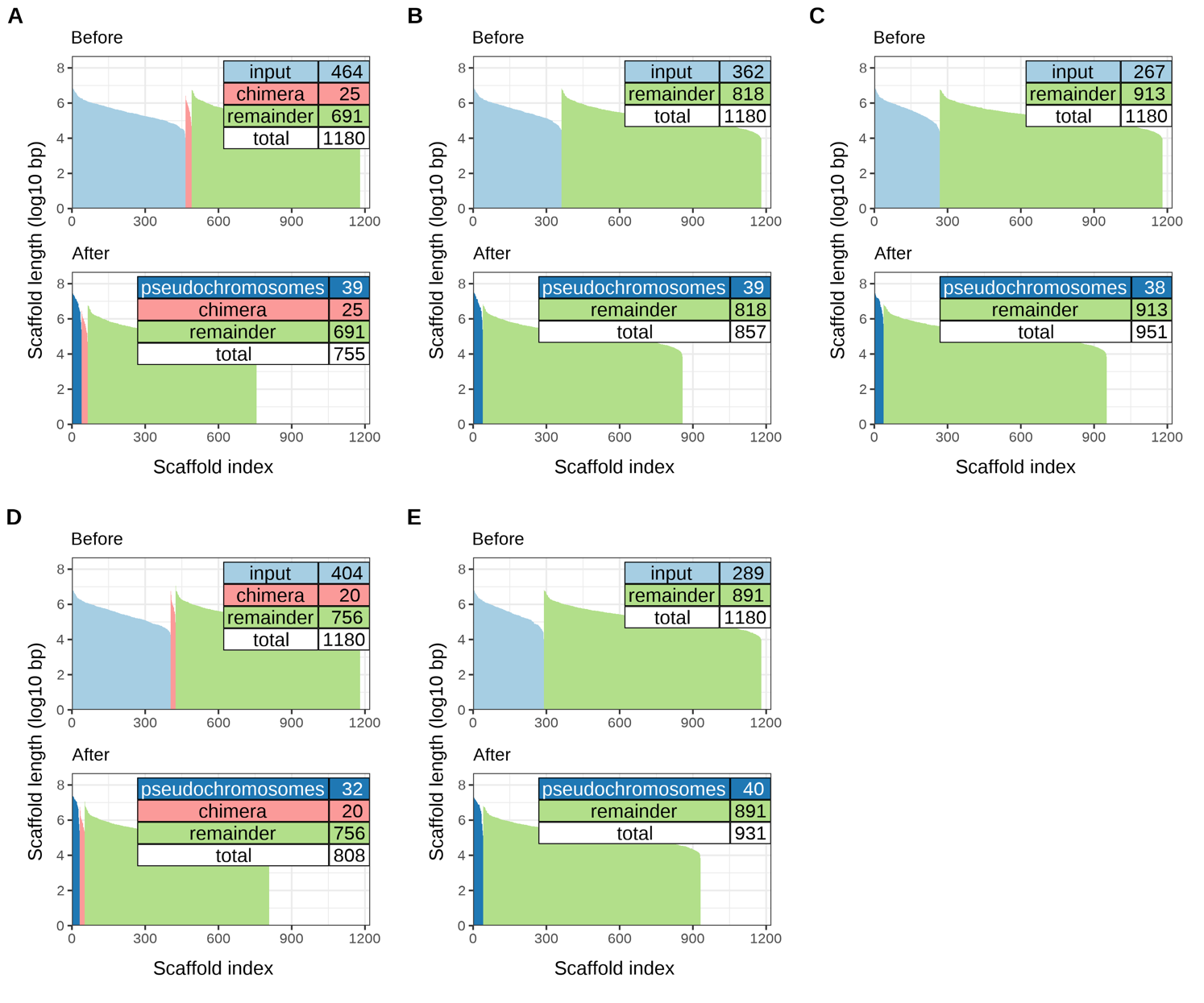


**Supplementary Figure 3.** Results of re-scaffolding applied to the v1 *Saccharina latissima* assembly with varying parameters given to Ragout: (A) no size filtering, chimeric assembly allowed, (B) no size filtering, unbroken input scaffolds, (C) no size filtering, unbroken input scaffolds, forced HAL instead of MAF, (D) short contigs filtered out of comparison genomes, chimeric assembly allowed, (E) short contigs filtered out of comparison genomes, unbroken input scaffolds.


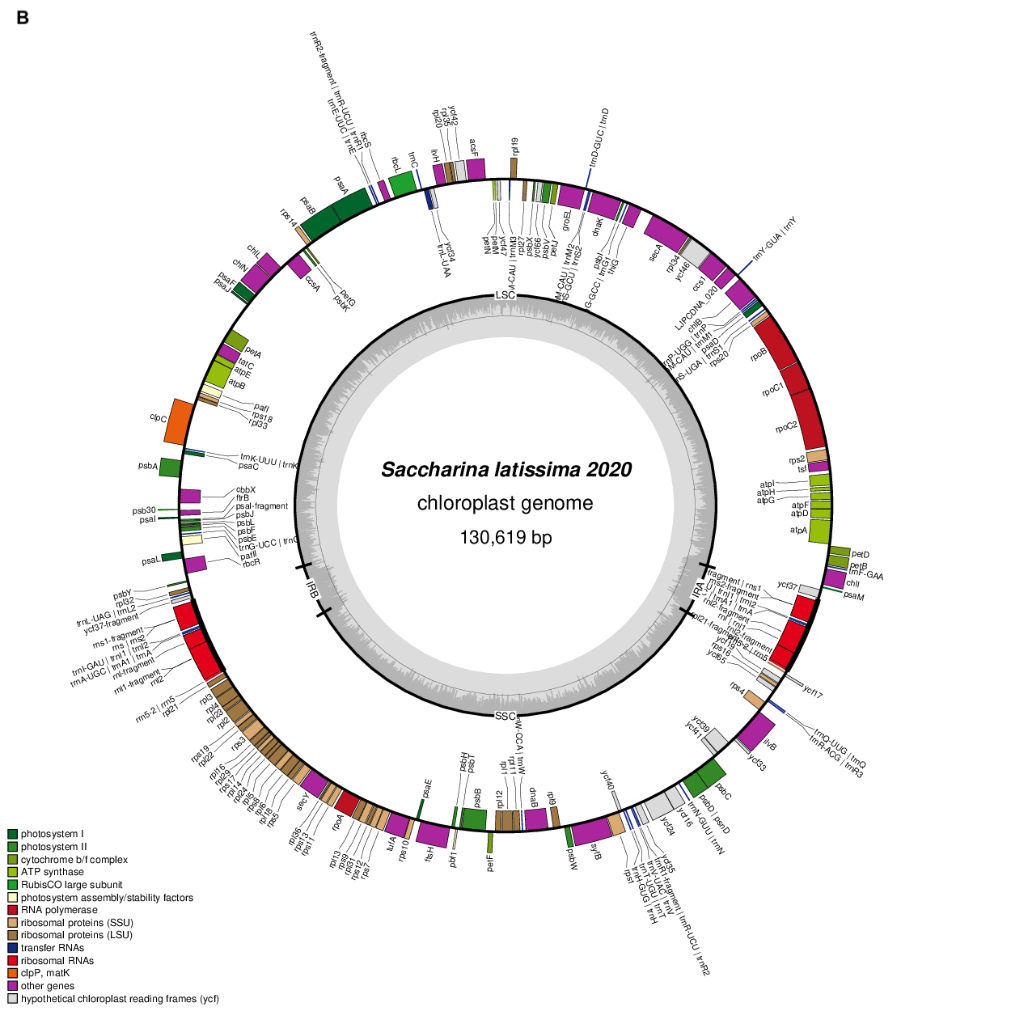


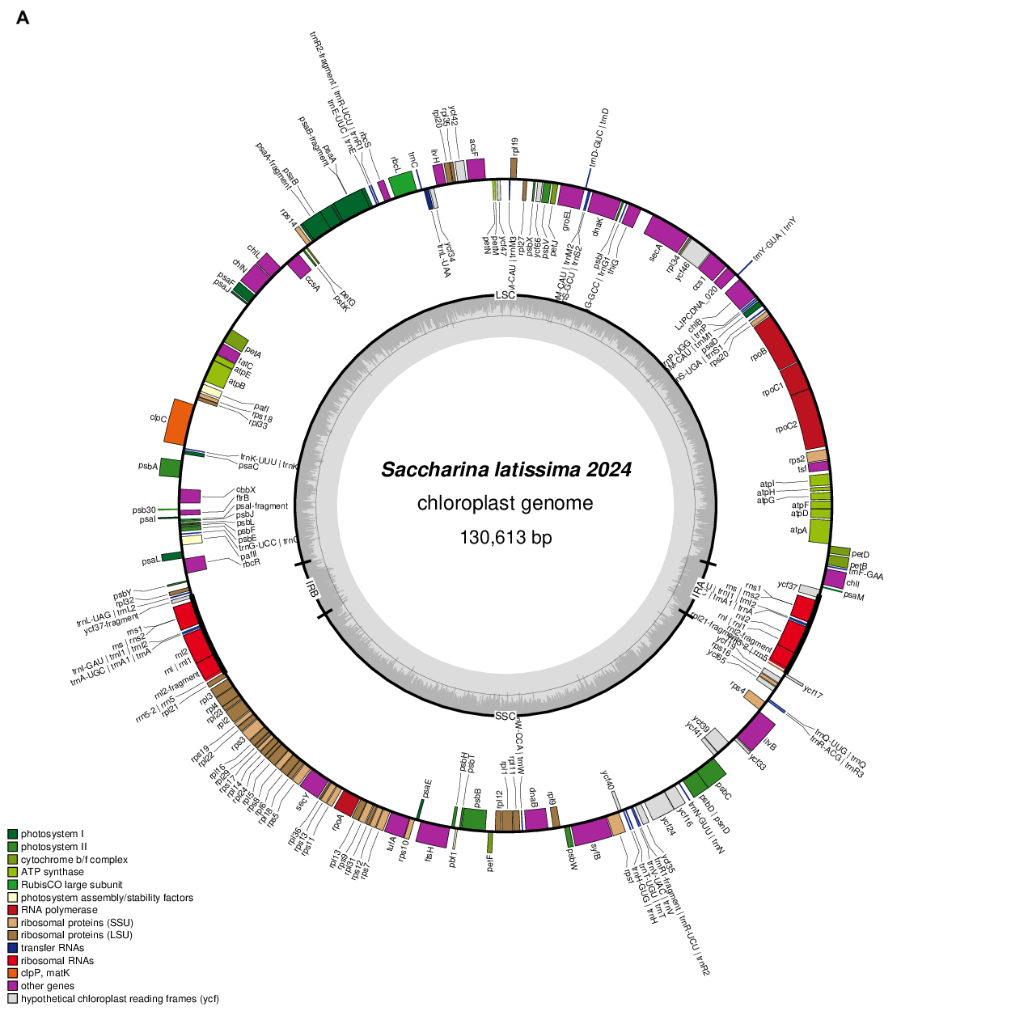


**Supplementary Figure 4.** Gene annotation of **(A)** this *Saccharina latissima* chloroplast genome assembly and **(B)** the previously published version.


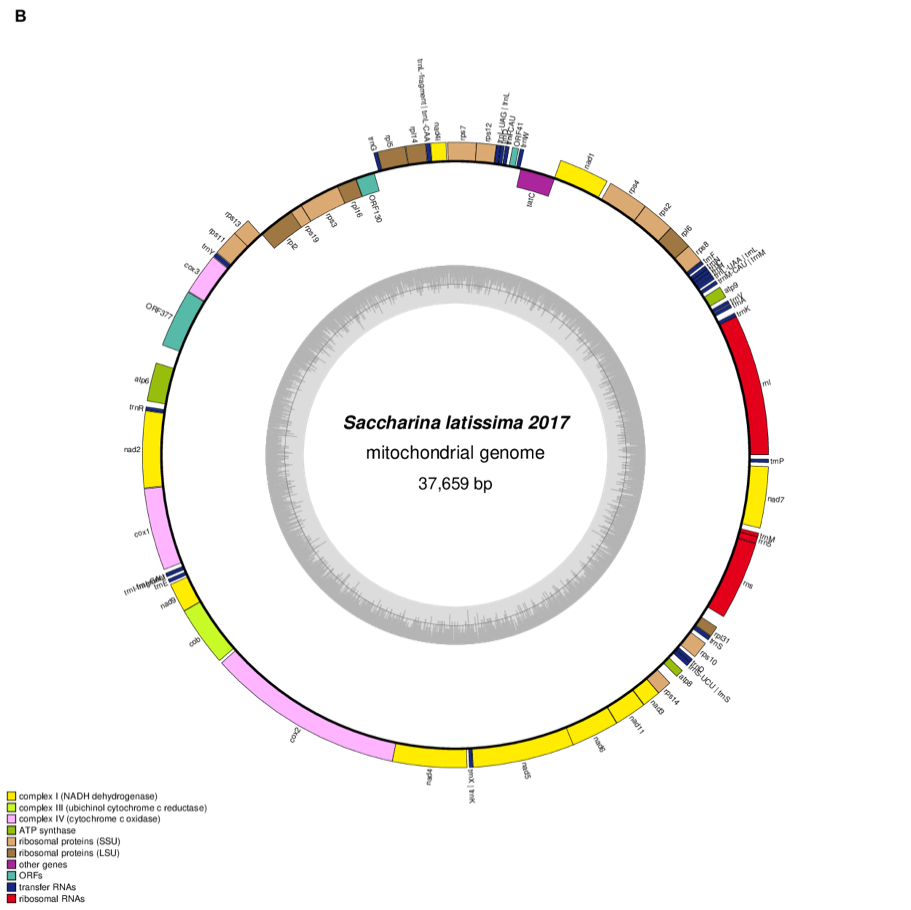

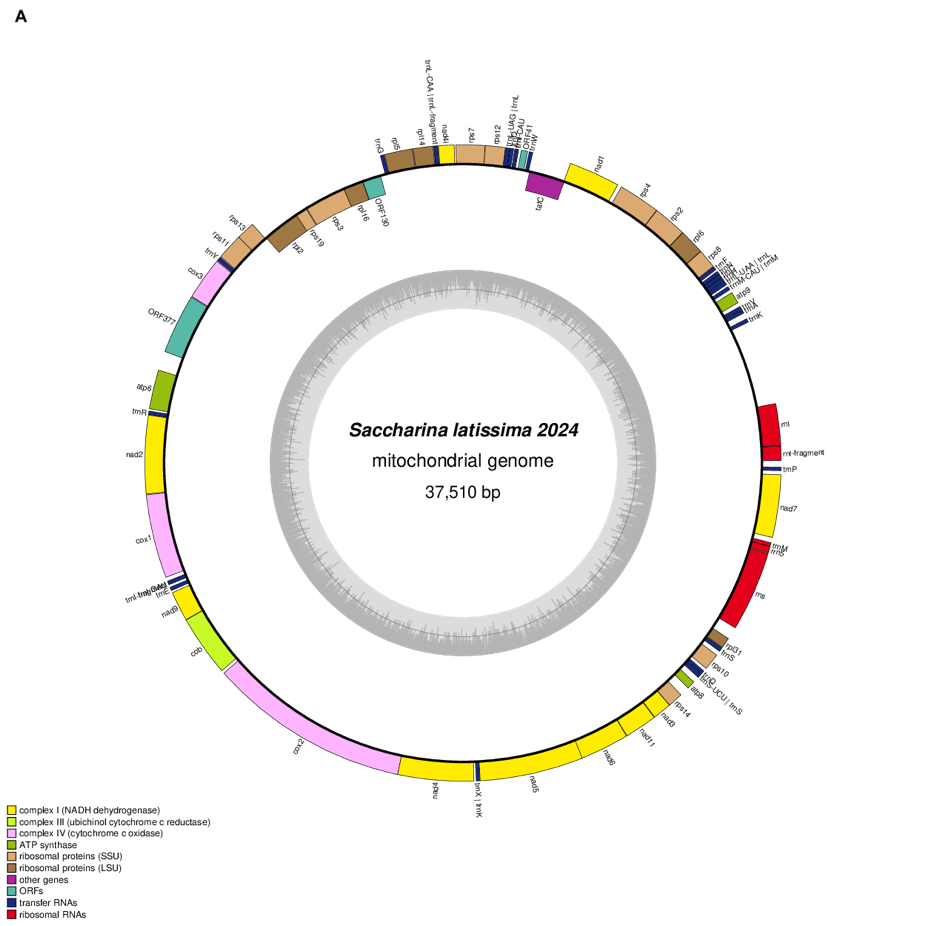


**Supplementary Figure 5.** Gene annotation of **(A)** this *Saccharina latissima* mitochondrial genome assembly and **(B)** the previously published version.


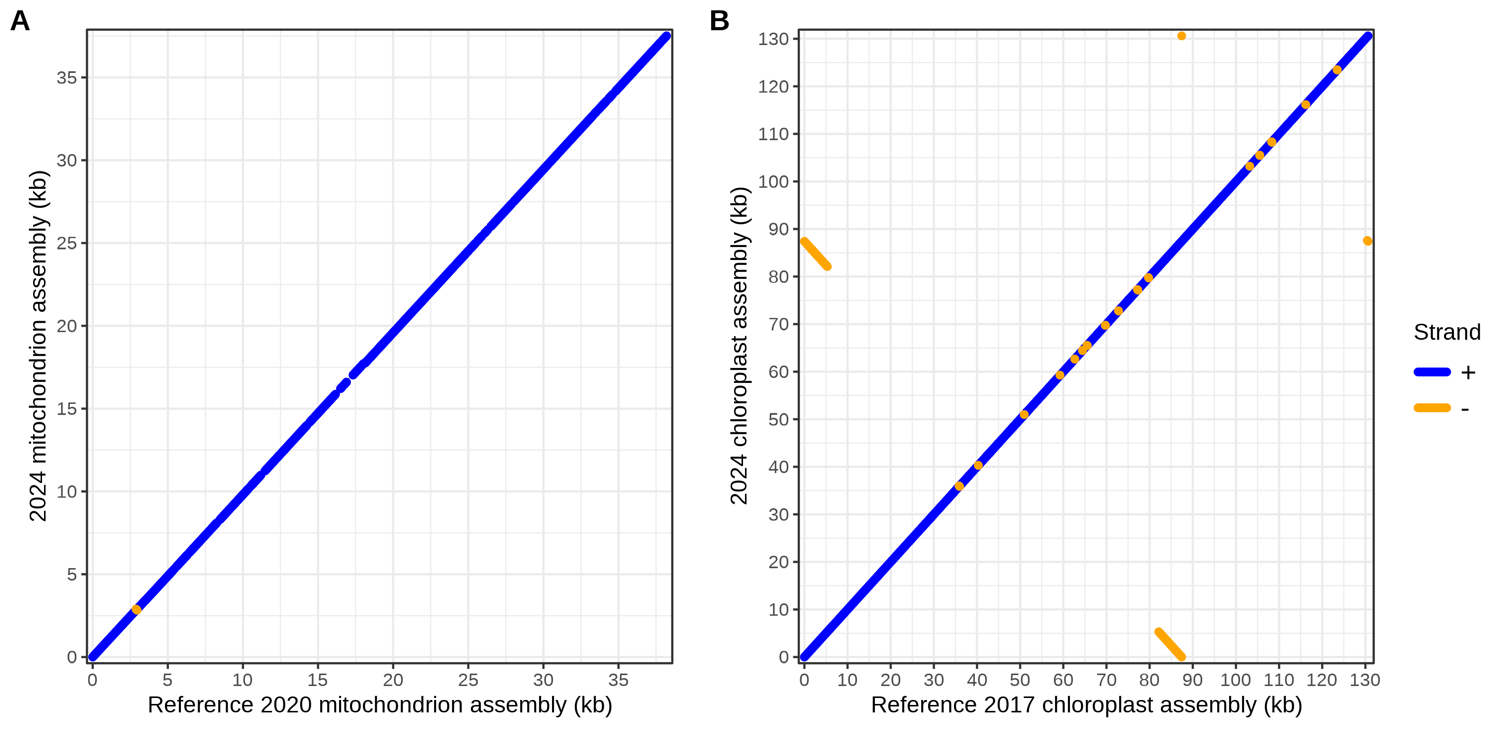


**Supplementary Figure 6.** Dotplots of **(A)** mitochondrion and **(B)** chloroplast genome assemblies aligned to previous versions. Segments that align in the forward direction (+) are blue, while segments that align along the complementary strand (-) are orange.

## Supplementary Tables

| **Library** | **Sequencing Platform** | **Average Read/Insert Size** | **Read Number** | **Assembled Sequence Coverage (x)** |
| --- | --- | --- | --- | --- |
| IYHH | Illumina (2x150) | 400 | 282,564,006 | 66.03 |
| JAZK | Illumina-HiC  (2x150) | N/A | 626,664,456 | 145.33 |
|  | PacBio | 8,389^*^ | 11,430,834 | 180.56 |
| **Total** |  | N/A | 920,659,296 | 391.92 |

**Table S1.** Genomic libraries included in the *Saccharina latissima* nuclear genome assembly and their respective assembled sequence coverage levels in the final release. ^*^Average read length of PacBio reads.

| **Cutoff** | **Number of Reads** | **Basepairs** | **Average Read Length** | **Coverage** |
| --- | --- | --- | --- | --- |
| 0 | 11,430,834 | 111,139,900,311 | 8,389 | 180.56x |
| 1,000 | 11,426,757 | 111,137,691,066 | 8,391 | 180.56x |
| 2,000 | 11,406,850 | 111,106,697,298 | 8,400 | 180.50x |
| 3,000 | 11,362,643 | 110,992,506,950 | 8,420 | 180.31x |
| 4,000 | 11,248,297 | 110,583,256,664 | 8,474 | 179.65x |
| 5,000 | 10,857,495 | 108,778,137,020 | 8,662 | 176.71x |
| 6,000 | 9,274,040 | 99,971,100,832 | 9,494 | 162.41x |
| 7,000 | 7,501,551 | 88,486,830,709 | 10,554 | 143.75x |
| 8,000 | 6,153,382 | 78,406,619,727 | 11,486 | 127.37x |
| 9,000 | 5,095,863 | 69,436,380,004 | 12,353 | 112.80x |
| 10,000 | 4,199,440 | 60,931,006,064 | 13,236 | 98.98x |
| 11,000 | 3,414,452 | 52,698,473,044 | 14,177 | 85.61x |
| 12,000 | 2,751,358 | 45,083,558,817 | 15,163 | 73.24x |
| 13,000 | 2,211,845 | 38,349,212,951 | 16,160 | 62.30x |
| 14,000 | 1,775,287 | 32,463,759,860 | 17,168 | 52.73x |
| 15,000 | 1,425,810 | 27,403,071,208 | 18,160 | 44.51x |
| 16,000 | 1,145,593 | 23,065,013,041 | 19,137 | 37.47x |
| 17,000 | 921,034 | 19,364,018,470 | 20,091 | 31.45x |
| 18,000 | 738,743 | 16,177,069,267 | 21,042 | 26.28x |
| 19,000 | 590,689 | 13,440,647,354 | 21,979 | 21.83x |

**Table S2.** PacBio library statistics for the libraries included in the *Saccharina latissima* nuclear genome assembly and their respective assembled sequence coverage levels.

| **Minimum**  **Scaffold**  **Length** | **Number of**  **Contigs** | **Scaffold Size** | **Basepairs** | **% Non-gap Basepairs** |
| --- | --- | --- | --- | --- |
| 5 Mb | 14 | 95,381,417 | 95,381,417 | 100.00% |
| 2.5 Mb | 47 | 219,364,104 | 219,364,104 | 100.00% |
| 1 Mb | 183 | 426,743,239 | 426,743,239 | 100.00% |
| 500 Kb | 387 | 572,290,709 | 572,290,709 | 100.00% |
| 250 Kb | 713 | 688,982,581 | 688,982,581 | 100.00% |
| 100 Kb | 1,368 | 793,335,385 | 793,335,385 | 100.00% |
| 50 Kb | 1,971 | 835,227,866 | 835,227,866 | 100.00% |
| 25 Kb | 4,090 | 910,555,146 | 910,555,146 | 100.00% |
| 10 Kb | 4,849 | 925,745,371 | 925,745,371 | 100.00% |
| 5 Kb | 4,853 | 925,781,601 | 925,781,601 | 100.00% |
| 2.5 Kb | 4,853 | 925,781,601 | 925,781,601 | 100.00% |
| 1 Kb | 4,854 | 925,783,470 | 925,783,470 | 100.00% |
| 0 bp | 4,854 | 925,783,470 | 925,783,470 | 100.00% |

**Table S3.** Summary statistics of the initial output of the RACON polished HiFiAsm+HIC *Saccharina latissima* nuclear genome assembly. The table shows total contigs and total assembled basepairs for each set of scaffolds greater than the size listed in the left-hand column.

| **Scaffold total** | 1,180 |
| --- | --- |
| **Contig total** | 1,513 |
| **Scaffold sequence total** | 615.5 Mb |
| **Contig sequence total** | 612.2 Mb (0.5% gap) |
| **Scaffold L50 / N50** | 109 / 1.4 Mb |
| **Contig L50 / N50** | 165 / 971.7 Kb |

**Table S4.** Final summary assembly statistics for the version 1.0 *Saccharina latissima* nuclear genome assembly.

| **Statistic** | ***Ectocarpus* sp. Ec32** | ***U. pinnatifida*** | ***M. pyrifera*** | ***S. japonica*** | **All** |
| --- | --- | --- | --- | --- | --- |
| **Average n homologous scaffolds mapped per chromosome** | 31.03 ± 16.71 | 35.09 ± 60.59 | 18.18 ± 15.5 | 31.57 ± 11.38 | 26.94 ± 31.23 |
| **Maximum n homologous scaffolds mapped per chromosome** | 60 | 362 | 57 | 74 | 362 |
| **Minimum n homologous scaffolds mapped per chromosome** | 1 | 6 | 1 | 16 | 1 |
| **Total n homologous scaffolds mapped** | 1024 | 1123 | 1091 | 884 | 4122 |
| **Average length of homologous scaffolds (Mb) per chromosome** | 17.77 ± 8.62 | 19.17 ± 20.77 | 10.11 ± 8.34 | 19 ± 6.95 | 15.28 ± 12.52 |
| **Maximum length of homologous scaffolds (Mb) per chromosome** | 34.66 | 126.18 | 28.4 | 35.04 | 126.18 |
| **Minimum length of homologous scaffolds (Mb) per chromosome** | 0.03 | 4.21 | 0.03 | 7.07 | 0.03 |
| **Total length of homologous scaffolds (Mb)** | 586.3 | 613.38 | 606.34 | 531.98 | 2338 |
| **Average exact matches (Mb) per chromosome** | 1.07 ± 0.74 | 5.68 ± 5.7 | 1.52 ± 1.39 | 0.62 ± 0.38 | 2.13 ± 3.31 |
| **Average exact matches (%) per chromosome** | 6.76 ± 3.58 | 41.14 ± 9.14 | 18.92 ± 9.56 | 9.32 ± 4.02 | 19.19 ± 14.52 |
| **Maximum exact matches (Mb) per chromosome** | 2.52 | 35.36 | 5.07 | 1.77 | 35.36 |
| **Minimum exact matches (Mb) per chromosome** | 0 | 1.68 | 0.01 | 0.21 | 0 |
| **Total exact matches (Mb)** | 35.36 | 181.89 | 90.93 | 17.24 | 325.43 |

**Table S5.** Summary statistics of *S. latissima* whole genome alignments per homologous chromosome in *S. japonica*, *M. pyrifera*, *U. pinnatifida*, and *Ectocarpus* sp. Ec32.
